# Examining the Performance of ChatGPT in Comprehensive Pre‐Internship Exam: The Potential of Artificial Intelligence in Medical Education

**DOI:** 10.1002/hsr2.71492

**Published:** 2026-01-08

**Authors:** Michaeel Motaghi Niko, Zahra Karbasi, Maryam Kazemi, Maryam Zahmatkeshan

**Affiliations:** ^1^ Department of Nursing Shahrekord University of Medical Sciences Shahrekord Iran; ^2^ Department of Health Information Sciences, Faculty of Management and Medical Information Sciences Kerman University of Medical Sciences Kerman Iran; ^3^ Noncommunicable Diseases Research Center Fasa University of Medical Sciences Fasa Iran; ^4^ School of Allied Medical Sciences Fasa University of Medical Sciences Fasa Iran

**Keywords:** artificial intelligence, ChatGPT, examination, medical education

## Abstract

**Background and Aims:**

ChatGPT is a popular large language model with potential educational applications in medicine. However, its performance in standardized, multi‐disciplinary medical exams has not been comprehensively assessed. This study evaluates ChatGPT's accuracy and quality in Iran's national medical pre‐internship exam.

**Methods:**

We tested ChatGPT (GPT‐3.5, May 3rd version) on 195 multiple‐choice questions from the March 2022 Iranian pre‐internship exam, covering 23 medical specialties. Questions with visual content were excluded. Each question was asked in a new chat to avoid memory bias. Responses were evaluated by 55 experts using a 5‐point Likert scale and compared against the official answer key. Data were analyzed descriptively using SPSS.

**Results:**

ChatGPT answered 68.6% of questions correctly. Expert ratings averaged 4.23/5 (SD = 1.21), indicating good to excellent quality. Best‐performing specialties included pharmacology (85.7%), otorhinolaryngology (83.3%), and dermatology (83.3%). Lower performance was observed in pulmonology (42.9%) and epidemiology (50%).

**Conclusion:**

ChatGPT shows promise as a supplemental educational tool in medical education, but its accuracy varies by specialty. Faculty guidance is essential to ensure responsible integration until further improvements and validations are made.

## 1 Introduction

The rapid advancement of artificial intelligence (AI) is ushering in a new era of technology‐driven transformation across numerous industries, including healthcare and medical education [1]. One of the popular and famous tools based on artificial intelligence is the Chat‐Generative Pre‐Trained Transformer (ChatGPT), which is considered a large language model (LLM) [2]. This Generative‐Pre‐Trained Transformer‐3.5 (GPT‐3.5)‐based chatbot is published by OpenAI [3] and it has 175 billion parameters and can provide human‐like answers to the user by using deep learning algorithms on a large amount of data. It was created by adding supervised and reinforcement learning techniques to the previous GPT‐3.5 language models [3]. ChatGPT, using a large collection of information including books, online websites, and articles leading to 2021, can provide comprehensible, fluent, and knowledge‐based answers in various fields, including medicine [4]. The use of artificial intelligence chatbots such as ChatGPT in the teaching and learning process can help students understand complex concepts, help in education and solve assignments. This chatbot can improve the learning process by providing accurate and fluent answers. However, there are also concerns that the widespread use of this chatbot by students may have negative effects on the learning process. This issue requires attention and proper management of the use of this chatbot [5]. This chatbot has been able to obtain promising results in admission tests in the UK, these tests included the medical admission test, the math test for university admission, and so on [6]. Also, it has passed the US Medical Licensing Exam without any specialized training [7]. Other studies have measured the performance of ChatGPT in the American Heart Association Life Support Test, Family Medicine Test, and Plastic Surgery Service Test [8, 9, 10]. Another study has been conducted on the performance of ChatGPT in the cardiology test [11], but as far as we know, no study has investigated the performance of this artificial intelligence in a comprehensive test with many different fields of medicine (23 fields).

Iranian medical pre‐internship test is used to evaluate the ability and competence of medical students in the clinical environment. This test usually includes multiple choice questions in 23 different fields of medicine, including seven different specialties of internal medicine, general surgery, pediatric, obstetrics and gynecology, neurology, infectious diseases, radiology, pathology, otorhinolaryngology (ear, nose and throat), medical statistics and epidemiology of common diseases, pharmacology, and medical ethics. In general, pre‐internship testing is used to ensure that students have the necessary abilities and skills to perform clinical tasks. This test can be effective as a useful tool in improving the quality of medical services and improving the ability level of medical students [12]. ChatGPT has the potential to be an effective tool for medical education [9]. According to the studies conducted so far, no study has evaluated the performance of ChatGPT in a multi‐part test from the perspective of experts. Therefore, the purpose of the research is to investigate the performance of ChatGPT in Iran's medical pre‐internship test and analyze the answers for the interpretability of users. This study is significant for several reasons. First, it evaluates the performance of ChatGPT in a comprehensive national medical exam that covers 23 different medical disciplines—something that has not been previously studied in this depth. Second, it incorporates both automated answer‐checking and human expert ratings, offering a more nuanced view of the chatbot's output quality. The findings can guide educators, policymakers, and developers in responsibly integrating AI tools into medical education. Given the growing reliance on large language models in education and healthcare, understanding their reliability and limitations is crucial.

## 2 Methods

### 2.1 Study Design

This was a cross‐sectional, observational study conducted to evaluate ChatGPT's performance on a comprehensive standardized medical exam. Iran's medical pre‐internship exam includes 200 single best‐answer multiple choice questions (MCQs), with four options each, that contains 46 internal medicine questions in the subcategories: six Rheumatology questions, seven Pulmonology questions, six Cardiology questions, seven Gastroenterology questions, six Nephrology questions, six Hematology questions, and seven Endocrinology questions, 24 General Surgery questions, 24 Pediatric questions, 19 Obstetrics and gynecology questions, eight Neurology questions, nine Infectious Diseases questions, six Radiology questions, nine Pathology questions, seven Psychiatric questions, seven Dermatology questions, seven Orthopedics questions, six Urology questions, six Ophthalmology questions, six Otorhinolaryngology (Ear, Nose and Throat) questions, six Medical statistics and Epidemiology of Common Diseases questions, seven Pharmacology questions, three questions are Medical Ethics. Since the last access of the ChatGPT database is until September 2021, to ensure that none of the answers, explanations, or related content in the ChatGPT data is available from the English language question book issued by the Testing Center for Medical Education on March 3, 2022 published were used. All survey questions and questions requiring visual analysis, including clinical images, medical images, and charts, were excluded from the study due to ChatGPT's inability to analyze image inputs and finally, 195 questions remained.

The official pass mark for the national pre‐internship exam is 120 out of 200 questions (60%). ChatGPT's performance was evaluated against this benchmark. No statistical adjustment was applied for excluded visual questions, and only text‐based questions were retained for consistency with ChatGPT's current capabilities. All ChatGPT responses were collected by two authors (MM, MZ), in the May 3rd edition of ChatGPT and independently verified for accuracy and consistency before statistical analysis. To reduce data retention bias in the chatbot's memory, each question was asked separately in a new chat and the responses were collected. The accuracy of ChatGPT responses was rated by 55 experts (at least two experts in each topic and some topics three or four experts) on a 5‐point Likert scale (from very poor/unacceptable to very good/no inaccuracy). Also, to measure the correct percentage of the answers, they were compared with the answer sheet published by the Ministry of Health of Iran to evaluate the accuracy. Finally, the data was analyzed statistically, percentage, average, and standard deviation were used to express the results. All analyses were conducted using IBM SPSS version 25 and tables and graphs were prepared using Microsoft Excel version 2019. Ethical approval for this study was obtained from the Ethics Committee of Fasa University of Medical Sciences (Approval ID: IR.FUMS.REC.1402.026). Expert evaluations were anonymized and voluntary. No personal data were used, and no patient or student consent was required as the study involved only secondary data.

### 2.2 Statistical Analysis

Statistical analysis was performed using IBM SPSS Statistics version 25. Descriptive statistics (percentage, mean, and standard deviation) were used. As this study primarily relied on descriptive metrics, no inferential statistical tests (e.g., *p*‐values) were used.

## 3 Results

Overall, ChatGPT achieved 68.6% correct answers, demonstrating fairly high. Based on the exam's official pass mark (60%), ChatGPT would be considered to have passed the exam. The mean expert rating was 4.23 (SD = 1.21), reflecting assessments of “good” to “excellent” overall quality.

In certain specialties, ChatGPT exhibited very high accuracy and ratings, such as in pharmacology (85.7%, mean = 4.38, SD = 1.12), otorhinolaryngology (ENT) (83.3% accuracy, mean = 4.92, SD = 0.29), ophthalmology (83.3%, mean = 3.92, SD = 1.68) and dermatology (83.33%, mean = 4.43, SD = 0.94).

In contrast, lower accuracy and more variability occurred in other domains like pulmonology (42.9%, mean = 3.64, SD = 1.55), Epidemiology (50.00%, mean = 3.25, SD = 1.71), endocrinology (57.1%, mean = 3.86, SD = 1.29) and gastroenterology (57.1%, mean = 4.07, SD = 1.21).

See Table 1 for full details of accuracy and expert ratings.

**Table 1:** ChatGPT's accuracy and expert ratings across medical specialties.

Specialty	Number of questions	Correct percentage	Mean experts rating	SD of experts rating
Pharmacology	7	85.71%	4.38	1.12
Otorhinolaryngology	6	83.33%	4.92	0.29
Dermatology	7	83.33%	4.43	0.94
Ophthalmology	6	83.33%	3.92	1.68
Pathology	9	77.78%	4.89	0.58
Infectious Disease	9	77.78%	4.22	1.31
Orthopedics	4	75.00%	4.75	0.46
Neurology	8	75.00%	4.19	0.66
Gynecology	19	73.68%	4.37	1
Psychiatry	7	71.43%	4.33	1.11
Pediatrics	23	69.57%	4.54	0.66
Ethics	3	66.67%	4.5	1.22
Radiology	6	66.67%	4.08	1.38
Rheumatology	6	66.67%	4.5	1.24
Cardiology	6	66.67%	4.38	1.28
Nephrology	6	66.67%	4.5	0.67
Hematology	6	66.67%	4	0.85
Urology	6	66.67%	4.39	0.85
General surgery	24	58.33%	3.67	1.63
Gastroenterology	7	57.14%	4.07	1.21
Endocrinology	7	57.14%	3.86	1.29
Epidemiology	6	50.00%	3.25	1.71
Pulmonology	7	42.86%	3.64	1.55
Total	195	68.60%	4.23	1.21

**Figure 1:**
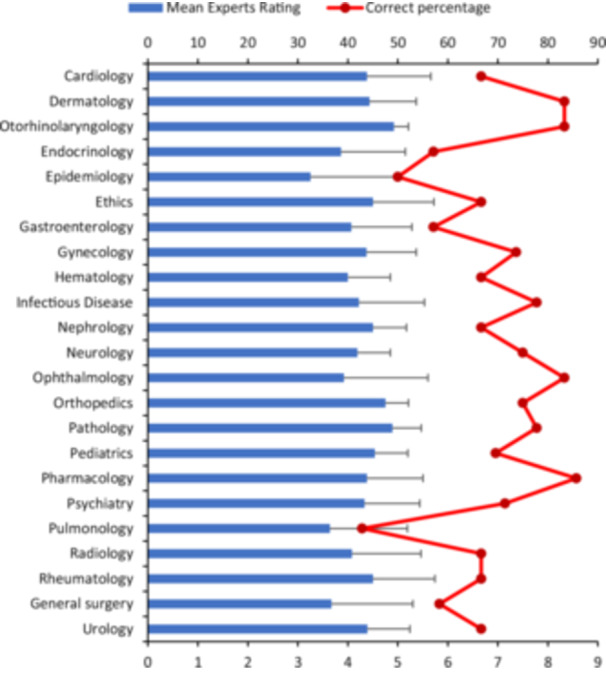
Performance of ChatGPT across medical specialties. This figure illustrates ChatGPT's performance on the medical pre‐internship exam, showing the percentage accuracy for each specialty along with expert ratings. Experts Ratings: The bar chart displays the experts ratings of ChatGPT's answers. The error bars represent the standard deviation of the ratings, calculated based on the variability of the expert scores. Accuracy of ChatGPT: The line graph shows the accuracy percentages for each medical specialty, with pharmacology (85.7%), otorhinolaryngology (83.3%), ophthalmology (83.3%), and dermatology (83.3%) showing the highest accuracy, while pulmonology (42.9%), epidemiology (50%), endocrinology (57.1%), and gastroenterology (57.1%) had lower accuracy.

See Figure 1 for a visual comparison of ChatGPT's performance across specialties.

The figure uses a red dashed line to represent accuracy percentages and blue bars to denote the average experts rating. The error bars are shown as black lines extending from each data point, indicating the standard deviation.

To manage the bias introduced by excluding image‐based questions, only domains where sufficient text‐based questions remained were included in the final analysis. No weighting adjustments were made, as all retained questions were considered equally representative within their specialty.

## 4 Discussion

Since the creation of ChatGPT, the investigation of its performance in the field of medical education has been increasing. Examining the performance of this chatbot in medical tests can clarify the weaknesses and strengths of artificial intelligence. This study was conducted with the aim of investigating the performance of ChatGPT in Iran's medical pre‐internship exam and analyzing the answers for the interpretability of users.

In this study, the accuracy of ChatGPT compared to the standard was 68.6%, which shows a relatively high performance. According to the average scores, the overall quality was good to excellent. Similar to our results, in Kung et al. study [13], which investigated the performance of ChatGPT on the medical licensing exam, the accuracy of its performance was above 50%. In Lai et al. study [14], ChatGPT achieved a good performance with a score of 76.3%.

ChatGPT demonstrated its highest expert rating in the field of otorhinolaryngology (mean = 4.92), indicating outstanding performance in this specialty. This suggests that in certain well‐defined clinical areas, ChatGPT's training data may sufficiently overlap with tested content, allowing for highly accurate and well‐reasoned answers. The high reliability in this domain points to the potential for using ChatGPT as a supplementary educational tool in medical learning. This means that ChatGPT could be used to help students self‐assess their knowledge by answering past exam questions and receiving immediate, interpretable responses. While it cannot replace human instruction, its ability to provide accurate answers in specific subjects may support revision and practice in medical education settings.

Beyond otorhinolaryngology, ChatGPT also achieved high expert ratings in orthopedics, pathology, pediatrics, and dermatology. These results suggest that while ChatGPT excels in certain specialties, its performance varies significantly across disciplines. This variability highlights both the opportunities and limitations of using generative AI models in medical education and underscores the need for field‐specific validation.

In the study of Al‐Shakarachi and Haq [15], who investigated the performance of ChatGPT in medical licensing, the results indicated that the accuracy of the chatbot in Otorhinolaryngology was 100%. Although the tests and questions in our study and their study were not the same, but achieving high accuracy in the specialty of Otorhinolaryngology can be considered. Contrary to our study, the performance of ChatGPT in [14] was slightly poor in the specialty of Otorhinolaryngology.

Several studies [13, 14, 16, 17, 18, 19, 20] investigated the performance of ChatGPT in medical tests. Ebrahimian et al. [20] state that ChatGPT performed exceptionally well in the Iranian medical licensing examination and was able to answer 68.5% of the questions correctly. Our study differs in several key aspects. We in addition to assessing the accuracy of ChatGPT's responses against the official answer key, we sought expert opinions from 55 medical specialists and subspecialists across various fields. As far as we know, this is the first study that examines experts' opinions about ChatGPT responses. This approach allowed us to obtain a more comprehensive evaluation of the quality and interpretability of ChatGPT's answers, as perceived by subject matter experts.

First, our study utilized the original English‐language version of the exam to ensure clarity and avoid translation bias. Second, we incorporated expert evaluations to assess the interpretability and reasoning quality of ChatGPT's answers. This eliminates potential inaccuracies or nuances that may arise from the translation process, ensuring that the questions were presented to ChatGPT in their original, intended form.

Furthermore, our research focused specifically on the Iranian medical pre‐internship examination, which encompasses a broad range (23 different fields) of medical disciplines and specialties. This multi‐disciplinary nature of the test allowed for a more comprehensive assessment of ChatGPT's capabilities across various medical domains. The performance of ChatGPT has been reported as acceptable in most of the mentioned studies, but in Weng et al. study [16], it did not have sufficient accuracy in answering the questions, which is probably related to factors such as the difficulty of the questions and the weak database. In our study, the lowest accuracy of ChatGPT performance was observed in the fields of surgery, pulmonology, and gastroenterology. It is possible that the questions in these specializations were more difficult. Another possible explanation for the variation in performance is the differential representation of domain‐specific content in ChatGPT's training data. Specialties like otorhinolaryngology and dermatology may be more prominently represented in publicly available English‐language medical texts and online content, while disciplines like surgery or pulmonology might include more context‐dependent clinical reasoning, or questions that are less well‐represented in pre‐2021 training data. However, without access to ChatGPT's proprietary training corpus, this remains speculative. In addition, the pre‐internship test questions may not be entered in the ChatGPT training database, and this will reduce the accuracy of searching and providing correct answers to the questions. Similar to the findings of [16], the mismatch between the data of the ChatGPT educational database and the test questions leads to the lack of access to information and incorrect answers.

The results of a meta‐analysis study [21] showed that ChatGPT showed acceptable performance in answering multiple‐choice questions. However, using this chatbot for medical tests should be done with caution so that it can be used as a reliable tool.

Just keep in mind that ChatGPT AI was trained with data from before 2021, so it is necessary to provide ChatGPT with more and more diverse resources such as texts, descriptions, articles, and research so that it does not run out of resources. For example, regarding the first question of medical ethics, in Iran, the legal age to decide on treatment is 18 years old, and before this age, parents must give their consent for treatment. In some cases, too, ChatGPT has been very clever. For example, in the second question of medical ethics, the reasoning type of ChatGPT is much more than a machine and it was able to choose the more correct option from two options that are close to each other.

Overall, although ChatGPT is impressive and effective, it has not yet reached the desired level of sophistication. Sometimes Chat GPT gives us an answer that is not actually true, but it presents this answer so convincingly that it is very difficult to tell if it is false. Only time will tell what the future of artificial intelligence will look like. But one thing can be said for sure; artificial intelligence is still in its infancy, but at this stage, it has shown capabilities that were not even imagined. In addition, ChatGPT shows promise in generating high‐quality clinical responses across medical domains. However, accuracy and consistency vary, indicating that further refinement of the AI system would be beneficial. Integrating physician collaboration and oversight with this technology could optimize strengths while minimizing limitations. Additional research on integrating ChatGPT into clinical workflows is warranted to fully evaluate its utility in improving medical decision‐making.

### 4.1 Limitations

To our knowledge, this is the first study to investigate the performance of ChatGPT in Iran's medical pre‐internship test and analyze the answers for the interpretability of users. This study includes several limitations. One limitation is that it is not clear whether ChatGPT has been tested on the multiple‐choice questions of the test. Some questions may not align with the logic of the chatbot, leading to decreased accuracy. Secondly, this study was specific to Iran's medical education system, so the results may not be generalizable to other regions. More research on ChatGPT's performance and the various factors influencing the answers is recommended. Additionally, we used ChatGPT version 3.5 in this study. Future studies can evaluate the capabilities of more advanced versions of this chatbot or other chatbots. We measured the experts' opinions on a 5‐point Likert scale (from very poor/unacceptable to very good/no errors), but this study did not include qualitative feedback from experts, which could have enriched the interpretation of ChatGPT's responses. Future studies should consider a mixed‐methods approach to capture nuanced expert perspectives.

## 5 Conclusion

ChatGPT has potential as a supplementary tool in medical education, but further development is needed. This means that although ChatGPT shows promise as a supplementary tool in medical education, further development is necessary to enhance its domain‐specific accuracy, reduce confidently incorrect outputs, and ensure alignment with curriculum standards. Improvements in transparency, reasoning depth, and error detection mechanisms would significantly improve its educational reliability. ChatGPT can generate credible medical information across different topics. However, ChatGPT sometimes provides false or inaccurate information in a convincing tone that makes it hard to discern misinformation. Thoughtfully integrating faculty guidance and oversight could help students utilize ChatGPT's capabilities while avoiding its flaws. Students can use ChatGPT to simulate clinical scenarios, clarify difficult concepts, or receive explanations for practice MCQs. While AI's future is uncertain, careful use of present‐day ChatGPT under faculty supervision may reveal constructive opportunities. With progressive improvements, ChatGPT could someday play a major role in medical education by complementing human teachers. Further research is required to find optimal ways to incorporate ChatGPT into medical school curricula and assessments, to fully evaluate its value in enhancing physician learning.

## Author Contributions

**Michaeel Motaghi Niko:** writing – original draft, conceptualization. **Zahra Karbasi:** writing – original draft, conceptualization. **Maryam Kazemi:** methodology, writing – original draft. **Maryam Zahmatkeshan:** conceptualization, writing – review and editing, supervision.

## Ethics Statement

This study was approved by the Ethics Committee of Fasa University of Medical Sciences (Approval ID: IR.FUMS.REC.1402.026). Since the study involved only secondary data without human subject participation or identifiable information, the requirement for participant consent was waived by the IRB. URL: https://ethics.research.ac.ir/ProposalCertificateEn.php?id=340770&Print=true&NoPrintHeader=true&NoPrintFooter=true&NoPrintPageBorder=true&LetterPrint=true.

## Conflicts of Interest

The authors declare no conflicts of interest.

## Transparency Statement

The lead author Maryam Zahmatkeshan affirms that this manuscript is an honest, accurate, and transparent account of the study being reported; that no important aspects of the study have been omitted; and that any discrepancies from the study as planned (and, if relevant, registered) have been explained.

## Data Availability

The data that support the findings of this study are available from the corresponding author upon reasonable request.
